# Genetic and phenotypic variation in wood tiger moths from the Caucasus: insights into male warning color variation

**DOI:** 10.1111/1744-7917.70101

**Published:** 2025-06-19

**Authors:** Juan A. Galarza, Ossi Nokelainen, Melanie N. Brien, Cristina Ottocento, Bibiana Rojas, Janne K. Valkonen, Tamar Chunashvili, Tõnis Tasane, Chris D. Jiggins, Johanna Mappes

**Affiliations:** ^1^ Ecology and Genetics Research Unit University of Oulu Oulu Finland; ^2^ Department of Biological and Environmental Science University of Jyväskylä Jyväskylä Finland; ^3^ Organismal and Evolutionary Biology Research Program, Faculty of Biological and Environmental Sciences University of Helsinki Helsinki Finland; ^4^ Department of Interdisciplinary Life Sciences, Konrad Lorenz Institute of Ethology University of Veterinary Medicine Vienna Vienna Austria; ^5^ Invertebrate Research Center, Tetritsklebi Telavi Georgia; ^6^ Fauna NPO Harju maakond Estonia; ^7^ Department of Zoology University of Cambridge Cambridge United Kingdom

**Keywords:** Arctiinae, color polymorphism, genetic structure, Georgia, Lepidoptera, multispectral imaging

## Abstract

Coloration plays a pivotal role in shaping how species adapt to their environment, influencing their interactions with predators, prey, and potential mates. The aposematic wood tiger moth (*Arctia plantaginis*) is sexually dimorphic. Males are polymorphic in their hindwing coloration across the Holarctic distribution range, while females exhibit continuous variation in their coloration. In the Caucasus region, a striking exception can be found, where both sexes exhibit a continuous orange‐red hindwing coloration. Yet, it remains uncertain whether significant color variations exist within the spectrum of male orange‐red coloration and whether these differences can be associated with genetic structure or other phenotypic traits such as size. Using population genetics and image analyses methods, we find that males from the Lesser Caucasus have predominantly large red wings and constitute mostly a single genetic population. Males from the Greater Caucasus, in contrast, appear genetically isolated and are relatively small with orange hindwings. We discuss these findings in the context of both contemporary and historical environmental factors that may have influenced male color variation in the region.

## Graphical abstract

Coloration serves several fitness‐related functions, including thermoregulation, immunity, social signaling, sexual selection, and predator avoidance. Consequently, color polymorphism can have a significant impact on a species’ interactions with its environment, including its relationships with predators, prey, and potential mates. The wood tiger moth (*Arctia plantaginis*) exhibits sex‐linked color polymorphism across most of its range: males display polymorphic hindwing coloration, while females consistently have an orange‐red coloration. An exception is found in the Caucasus region, where males appear to show a continuous gradient of orange‐red hindwing coloration. However, it is unclear whether substantial color variation exists within this gradient and if this variation corresponds to genetic and/or geographical features. Here, we employ image and genetic analyses to explore these relationships.

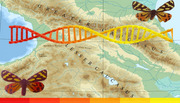

## Introduction

Coloration has multiple fitness‐linked functions such as thermoregulation (Stuart‐Fox *et al.*, 2017), immunity (Freitak *et al.*, 2005), social signaling (Tibbetts *et al.*, 2017), sexual selection, and predator avoidance (Ruxton *et al.*, 2018). Studies of color polymorphism have been useful in the study of evolution because they allow measuring selection on tractable phenotypic variation (Brakefield & Liebert, 1985). Color polymorphism can enhance population‐level performance and adaptability to novel or fluctuating environments, which can, in turn, lead to range expansion and decreased risk of extinction (Takahashi *et al.*, 2014; Ducatez *et al.*, 2017). In species that signal their unpalatability through warning coloration (aposematism), their coloration is predominantly genetically determined, ensuring consistent and effective predator‐deterrent signals crucial for survival (Joron *et al.*, 2011; Ottocento *et al.*, 2024; Koch *et al.*, 2025, but see Liu *et al.*, 2022). Thus color polymorphism can greatly affect a species’ interactions with its environment, predators, prey, and mates, and can be found in a wide range of taxa including plants and animals (Gray & McKinnon, 2007; Narbona *et al.*, 2017).

The wood tiger moth (*Arctia plantaginis*) (L., 1758) (Lepidoptera: Erebidae: Arctiinae) is an aposematic species widely distributed across the Holarctic (Hegna *et al.*, 2015). Both sexes advertise unpalatability through their hindwing coloration (Lindstedt *et al.*, 2011; Rojas *et al.*, 2019) which varies geographically (Hegna *et al.*, 2015). Male hindwing coloration shows discrete color polymorphism being yellow or white in Europe, Western Siberia and part of North America, and white or black in East Asia and North America (Hegna *et al.*, 2015). The yellow pigment is partially produced by pheomelanin, while the black coloration is derived from dopamine‐based eumelanin (Brien *et al.*, 2023). Yellow and white males co‐occur at different frequencies depending on the location (Galarza *et al.*, 2014; Rönkä *et al.*, 2020b) and differ in several fitness traits. Yellow morphs show stronger lytic responses (i.e., humoral immune response) (Nokelainen *et al.*, 2013). White morphs have greater mating success (Nokelainen *et al.*, 2012; Gordon *et al.*, 2015), and sustain longer flight activity (Rojas *et al.*, 2015). Both color morphs function as warning signals against bird predators, though the yellow morph is more conspicuous (Nokelainen *et al.*, 2012) and often has an advantage against bird and ant predators (Rojas *et al.*, 2017). However, despite being less conspicuous, the white morph experiences fewer predator attacks overall (Rojas *et al.*, 2019). Increased wing melanization provides thermoregulatory benefits, enhancing heat absorption, but also increases predation risk in both morphs (Hegna *et al.*, 2013). In contrast, female hindwing coloration shows a yellow‐red gradient throughout the species’ distribution (Hegna *et al.*, 2015). Experimental evidence shows that the orange‐red coloration has the highest efficacy as a warning signal (Lindstedt *et al.*, 2011; Rönkä *et al.*, 2018a).

**Fig. 1:**
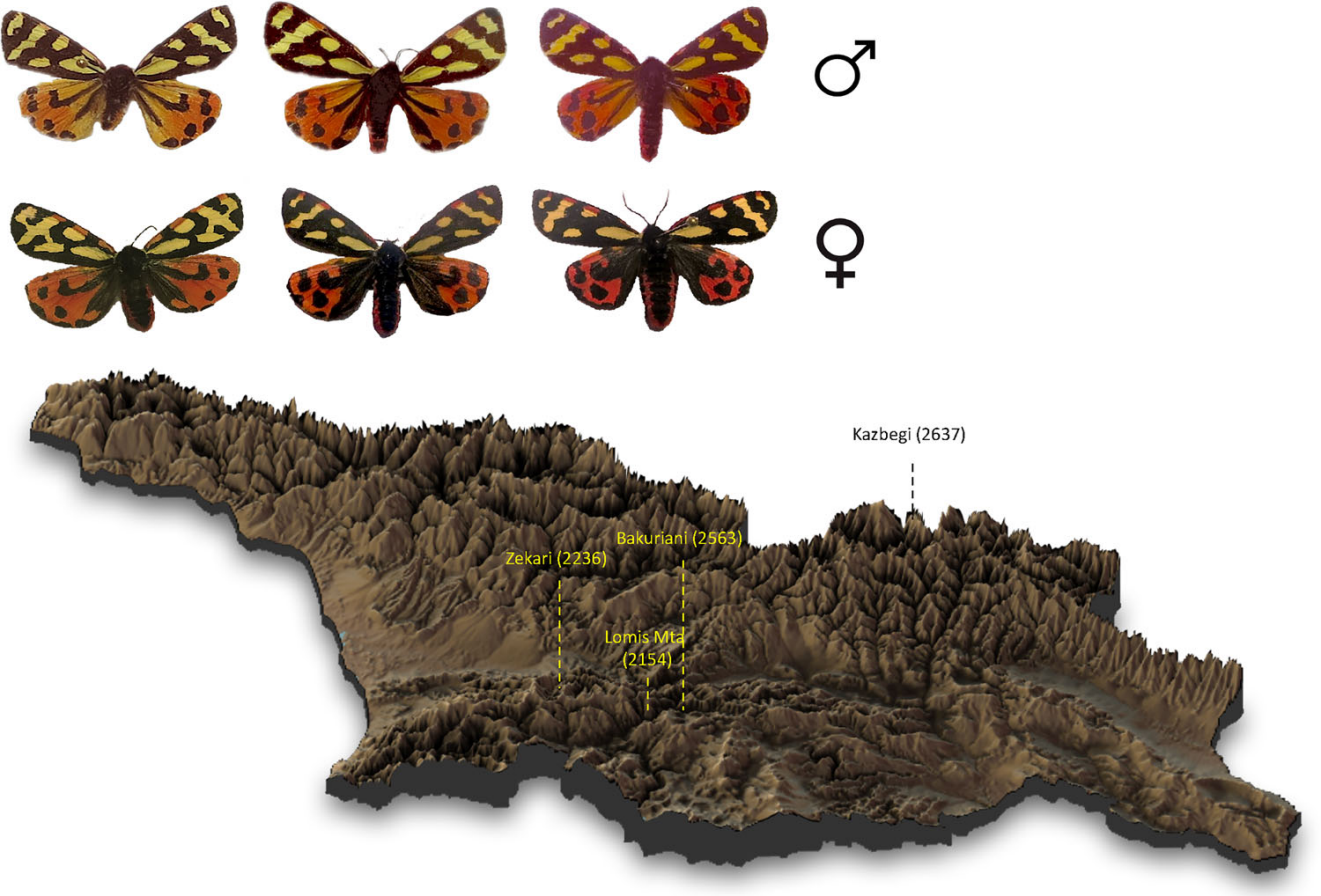
Wood tiger moth (*Arctia plantaginis*) males and females from Georgia. Orographic map of Georgia showing the sampling locations with elevations in meters above mean sea level indicated in parenthesis.

Two notable exceptions to sex‐limited color polymorphism exist in the wood tiger moth. One in Scotland, where both males and females exhibit uniform yellow‐orange hindwing coloration, and another in the Caucasus region, where both sexes display a continuous variation in orange‐red hindwing coloration (de Freina, 1993; Hegna *et al.*, 2015) (Fig. 1). In Georgia, the focal area of this study, it is not known if, and to which extent, significant color differences exist within the orange‐red color gradient observed in males. We have shown that chromatic diversity within white male morphs is imperceptible to human observers, while it may be visible to predators and mates (Nokelainen *et al.*, 2022). Thus, modern image analyses methods are necessary to objectively quantify color variation within the orange‐red gradient that may be of biological relevance.

The main aim of this study is to investigate if significant variation exists in the orange‐red male coloration and if such variation can be associated to genetic population structure in the Caucasus. To this end, we first characterize color variation in the fore‐ and hindwings of males from Georgia's Lesser and Greater Caucasus mountains using quantitative image analyses. We then identify contemporary and historical patterns of genetic variation and determine whether shifts in genetic structuring match color differences revealed by the image analyses. We hypothesize that significant variation exists in the extent of orange versus red observed in male hindwings, and that this variation segregates geographically. Alignment between coloration and genetic differences could suggest geographical isolation and drift as primary drivers of color divergence (i.e., redder than orange, or vice versa). Alternatively, homogeneity in coloration could be partly explained by current gene flow and/or historical connectivity. We also examine variation in wing size and perform whole‐genome analyses to investigate its potential genetic underpinnings.

## Materials and methods

### Sample collection

A total of 134 adult male wood tiger moths were collected in July 2016 across four sampling regions in Georgia for image and genetic analyses. Three regions (Zekari, Lomis Mta, and Bakuriani) are located in the Lesser Caucasus and one (Kazbegi) in the greater Caucasus (Fig. 1). The Greater Caucasus is characterized by high altitudes, rugged terrain, humid climate, whereas the Lesser Caucasus is defined by drier climate, and ecosystems adapted to semi‐arid conditions. Additionally, 12 more males were collected in Zekari in 2020 for whole‐genome analyses. All moths were collected using hand‐held butterfly nets by walking alongside mountain roads and trails during the day (10:00–17:00) in early July, which corresponds to peak flight season. The samples were individually stored in plastic containers at 4 °C in field station refrigerators before being transported to the lab, where they were kept at −20 °C until further processing.

### Coloration analyses

Of the total samples collected in 2016, only 70 were suitable for image analysis: Bakuriani (*n* = 13), Kazbegi (*n* = 19), Lomis Mta (*n* = 17), and Zekari (*n* = 30). Some samples were excluded due to damaged or broken wings with lost scales, which affected their coloration. The samples were meticulously pinned with their wings fully extended and photographed under controlled lighting conditions. Initial image calibration and analysis broadly followed previously used methods (Troscianko & Stevens, 2015; van den Berg *et al.*, 2020). Briefly, images were taken with a Samsung NX1000 digital camera converted to full spectrum (without quartz) and fitted with a Nikon EL 80 mm lens. A UV and infrared (IR) blocking filter (Baader UV/IR Cut Filter) was placed in front of the lens, allowing transmission of wavelengths between 400–680 nm. A standard light source, Exo‐Terra Sunray, which mimics sunlight across the spectrum, was used. For image calibration, gray reflectance standards (7% and 93% reflectance between 300–750 nm) were included. Calibration was performed using the Multispectral Image Calibration and Analysis (MICA) toolbox in ImageJ (Schneider *et al.*, 2012).

For reflectance data, we used normalized camera responses (i.e., no vision modelling) of red (R), green (G), and blue (B). For color analysis, the RGB channel values were converted to the HSV (i.e., hue, saturation, and brightness) color space to better characterize color space properties. Plainly, hue describes the dominant wavelength or “pure color” (e.g., a value that correlates with red or orange), saturation describes the intensity of color (i.e., expressed as the degree to which it differs from white), and brightness value describes how light or dark the object is. Finally, to assess for differences is wing size, we obtained the wing area using ImageJ by extracting the number of pixels from each image of the fore‐ and hindwing dorsal area and convert them to cm^2^.

### Statistical analyses

Due to the nonnormality of our data, we performed a Kruskal–Wallis test (one‐way ANOVA on ranks) to assess color and wing size variation across all sampling locations. Hue, saturation, and brightness metrics were used for color data, and wing area as a measurement of size. We then performed *post hoc* tests (Dunn's test) with false discovery rate correction for multiple testing to identify differences between pairs of locations. All analyses were conducted using R, version 3.5.0 (R Development Core Team, 2017).

### Contemporary demography analyses

A total of 134 individuals from 2016 (all regions) were genotyped at 9 nuclear microsatellite loci developed for the wood tiger moth (Galarza *et al.*, 2011) (DNA Processing). These included the 79 individuals from the coloration analysis above. Allele scoring was done by binning allele raw sizes using Tandem2 (Matschiner & Salzburger, 2009). We then used FreeNA (Chapuis & Estoup, 2007) to test the possibility of null alleles in each locus within sampling sites. Deviations from Hardy–Weinberg expectations (HWE) and linkage disequilibrium were then estimated according to the level of significance determined through 10 000 MCMC iterations using GENEPOP v.3.4. (Raymond & Rousset, 1995). Bonferroni corrections were applied for multiple comparisons setting an initial significance level of 0.05 (Rice, 1990).

We used different approaches to determine the contemporary population genetic structure. First, we used Structure v.2.3 (Pritchard *et al.*, 2000) to estimate the most likely number of genetic populations (*K*) present in our dataset. We launched the algorithm setting *K* from one to four, using the admixture model with correlated allele frequencies. We included all samples with no prior information about the sampling site or pre‐defined grouping. The algorithm was run for three million MCMC steps discarding the first third as burn‐in over five independent iterations. The *ad hoc* method of (Evanno *et al.*, 2005) was implemented to assess the likelihood of the different *K*s using STRUCTURE HARVESTER (Earl & vonHoldt, 2012). The software package CLUMMP v.1.1.2 (Jakobsson & Rosenberg, 2007) was used to evaluate the consistency of the results across the five iterations setting the full‐search algorithm. Second, we inferred the distribution of genetic variance through a principal component analysis (PCA) in the R package adegenet v.2.0 (Jombart, 2008). Allele frequencies were centered and scaled before calculating the principal components and the amount of variance represented by each. No grouping was defined prior to the analysis. We estimated FST (Weir & Cockerham, 1984) between all sampling pairs as implemented in hierfstat v0.04‐22 (Goudet, 2005), and assessed for demographic independence between sampling sites using the program BayesAss v.3.04 (Wilson & Rannala, 2003). Migrant ancestries of individuals were calculated through 10 million MCMC iterations with a burn‐in of three million, ensuring the mixing parameters *deltaA*, *deltaF*, and *deltaM* had an acceptance rate between 40% and 60% after the run. Convergence was assessed by examining the trace files of three independent runs using Tracer v.1.7.1 (Rambaut *et al.*, 2018).

### Historical demography analyses

We sequenced 630 bp from the cytochrome oxidase c subunit I (COI) gene using standard laboratory techniques (Supporting information DNA Processing) from the same individuals used in the microsatellite analyses (2016 samples, all regions, *N* = 174). The total number of distinct COI mtDNA haplotypes and their diversity (*H*) were determined in DnaSP v6 (Rozas *et al.*, 2017). A minimum spanning network was constructed in PopART (Leigh & Bryant, 2015) to examine the relationships among the mitochondrial haplotypes. To explore the historical demography of the four sampling sites, we analyzed the distribution of the observed pairwise nucleotide‐site differences (i.e., mismatch distribution) and the expected values for populations at equilibrium (i.e constant population size), and for growing or declining populations using Arlequin v3.1 (Excoffier & Lischer, 2010). The Harpending's raggedness index (*r*) (Harpending, 1994) was used to evaluate the fit of the observed distribution to the models via 10 000 bootstrap permutations.

### Genomic analyses

Genomic DNA was extracted from one or two legs of the Zekari samples (*N* = 12) and sequenced using an Illumina HiSeq 3000 at the Friedrich Miescher Laboratory, Tübingen, Germany, as part of a larger project. This data was analyzed to explore the genetic factors that may have contributed to the significantly larger wings observed in individuals from Zekari (see results). Although genome data from all sampling regions would have been ideal for a more effective comparative analysis, the data from Zekari is informative. By analysing signatures of selection such as reduced nucleotide diversity (*π*), negative Tajima's *D*, and high linkage disequilibrium (LD) we can identify genomic regions where selective sweeps have likely occurred (Vitti *et al.*, 2013). These genomic regions are strong candidates for containing genes that likely contribute to larger wings in this population. Furthermore, the identification of such regions can guide future comparative studies including data from other populations with smaller wings, providing a more detailed understanding of the genetic basis of wing size variation.

FASTQ reads were mapped using bwa‐mem (Li & Durbin, 2010) to the *Arctia plantaginis* scaffold‐level genome assembly (Yen *et al.*, 2020) available at NCBI BioProject: PRJEB36595. The resulting BAM files were sorted and indexed using SAMtools v.1.9 (Li *et al.*, 2009) and duplicates removed using PicardTools MarkDuplicates (broadinstitute.github.io/picard). The samples had an average read alignment of 93% and coverage of 8.75. SNPs were called using bcftools *mpileup* and *call* functions (Danecek *et al.*, 2021) with minimum mapping quality of 30. The VCF was filtered for depth (minDP 4, maxDP 15) allowing maximum missing data of 0.85, leaving a final set of 1 928 894 SNPs. Nucleotide diversity (*π*) was first calculated using a VCF that included invariant sites, produced and filtered as described above but without the *‐v* option (output variants only) in the bcftools *call* function. The software pixy (Korunes & Samuk, 2021) was used to estimate *π* across 20 Kb windows. Tajima's *D* was then calculated within the same 20 Kb windows using the variant‐only VCF in VCFtools (Danecek *et al.*, 2011). Following this, linkage disequilibrium (LD) was calculated for scaffolds identified with negative Tajima's *D* using VCFtools *–geno‐r2* function using one site every 10 Kb. To identify candidate genes within the genomic regions flagged by these analyses, we used the *A. plantaginis* white genome annotation (Yen *et al.*, 2020) to extract the coding sequences of genes within the windows, and performed a blastx (Camacho *et al.*, 2009) search against all *Drosophila melanogaster* proteins in FlyBase (v.FB2024_03, flybase.org/blast). All genome data generated here is freely available at the Sequence Read Archive of NCBI with accession number PRJNA1223277.

## Results

### Coloration and wing size results

**Fig. 2:**
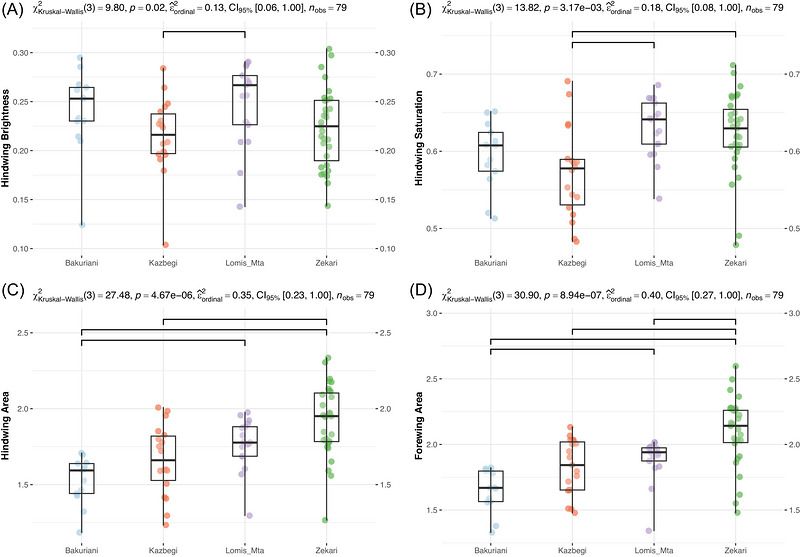
Male wood tiger moth (*Arctia plantaginis*) color metrics, including hindwing brightness (A), saturation (B), and wing areas (C, D). Brightness and saturation indicate the lightness and purity of the color respectively (range from 0 to 1). The wing area is shown in square centimeters. Pairwise *P*‐values of the *χ* ^2^ statistics after false discovery rate correction are denoted above brackets.

Significant differences were detected within the hindwing orange‐red gradient as indicated by the saturation values (*χ* ^2^ = 13.82, *P* ≤ 0.001; Fig. 2B). Specifically, individuals from Kazbegi exhibited the most pronounced orange‐yellow hues indicated by the lowest saturation values stemming from an increased proportion of middle wavelength reflectance. The lowest wing saturation of Kazbegi moths was followed in increasing order by Bakuriani and Zekari individuals. In contrast, Lomis Mta specimens exhibited the most pronounced red hues, indicative of a higher prevalence of longer wavelength reflectance (Fig. 2B). A similar trend was observed in the hindwing brightness (*χ* ^2^ = 9.80, *P* = 0.02), where individuals from Lomis Mta and Kazbegi show the brightest and dullest coloration, respectively (Fig. 2A). None of the other color metrics from the fore‐ or hindwings showed significant differences among localities (Fig. S1). The measurements of forewing size indicate that individuals from Zekari possess significantly larger wings compared to those from other populations (*χ* ^2^ = 30.90, *P* < 0.001). The same difference in wing size is also evident in hindwing, where individuals from Zekari generally have larger wings, except for those from Lomis Mta, which display similar hindwing sizes (*χ* ^2^ = 27.48, *P* < 0.001) (Fig. 2C, D).

### Contemporary demography results

**Fig. 3:**
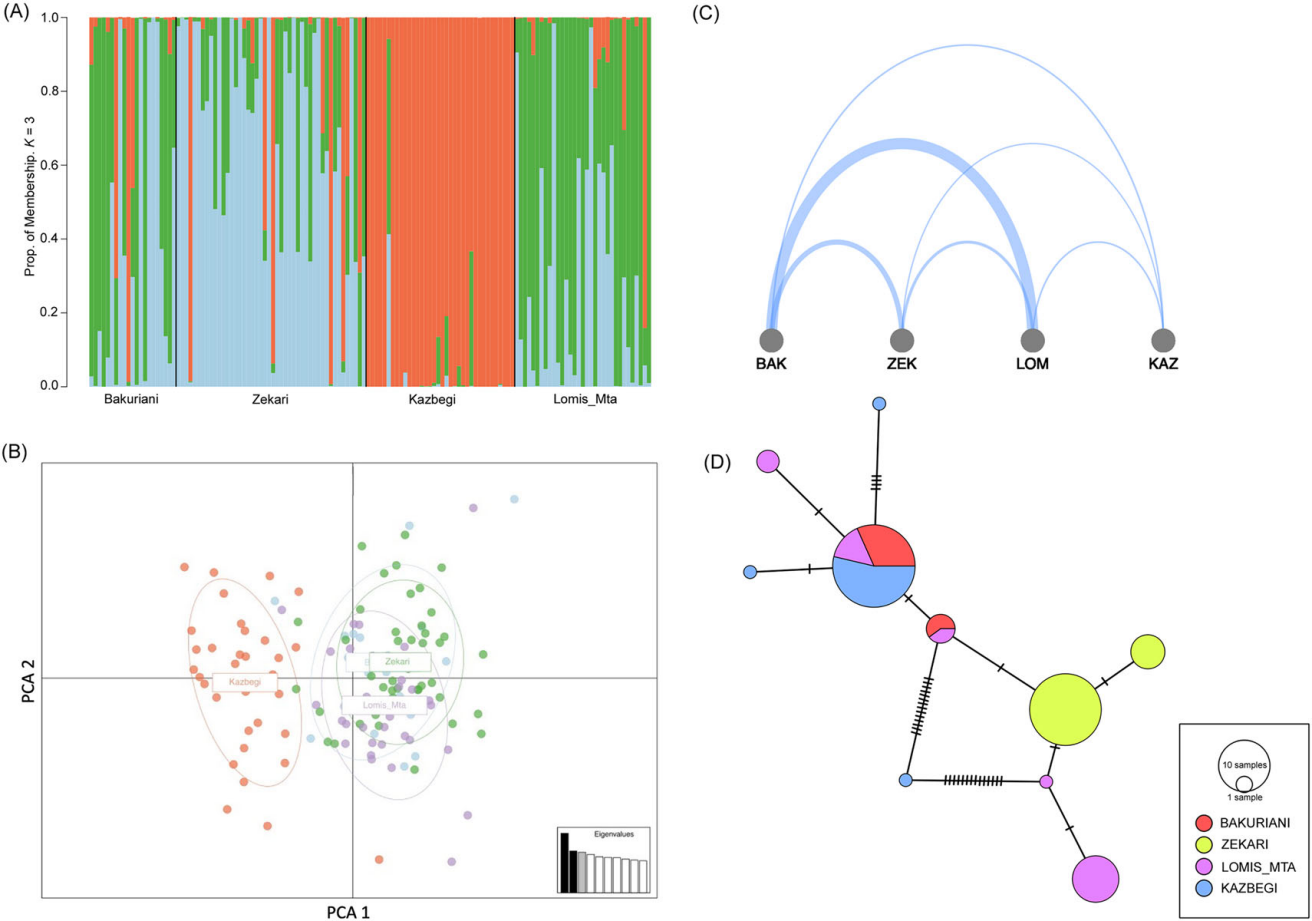
(A) STRUCTURE analysis of probability of population membership assuming three populations (*K* = 3). (B) Principal component analysis of allele frequencies from individuals of the different sampling sites. (C) Means of posterior probabilities of migration rates (*m*) between individuals of the different sampling sites. The thickness of the connecting arcs is proportional to *m* (see Table 2). (D) Mitochondrial COI minimum spanning network showing the relationship among haplotypes observed in the different sampling sites. Mutational steps are represented with hatch bars.

Microsatellite summary statistics are presented in Table S1. The results from the STRUCTURE analysis indicated that the most likely number of genetic groups present in our dataset (*K*) is three (Fig. 3A). Most samples from Kazbegi were clearly assigned to the same genetic group, followed by samples from Zekari being assigned less clearly to a different genetic group. Samples from Lomis Mta and Bakuriani had mixed assigned probabilities (Fig. 3A). The PCA analysis showed samples from Kazbegi forming their own cluster, whereas samples from the other three sampling sites could not be clearly separated (Fig. 3B). Pairwise *F_ST_* estimates showed significant differentiation only between Kazbegi and the rest of the sampling sites (Table 1). The Bayesian analyses of demographic independence showed that individuals from Bakuriani and Lomis Mta had the highest contemporary migrant ancestry between them, followed by individuals of Zekari, whereas individuals from Kazbegi had the least shared migrant ancestry with the other samples (Fig. 3C and Table 1).

**Table 1:** Sampling sites pairwise *F_ST_* comparisons

Site 1	Site 2	*Fst*	*m*	*Dist* (km)
Zekari	Bakuriani	0.022	0.0245	56.26
Kazbegi	Bakuriani	0.111*	0.0082	136.64
Lomis Mta	Bakuriani	0.020	0.0532	26.79
Kazbegi	Zekari	0.141*	0.0067	173.75
Lomis Mta	Zekari	0.044	0.0138	32.38
Lomis Mta	Kazbegi	0.101*	0.0081	143.79

*Note*: Means of posterior probabilities of migration rates (*m*) after 10 million MCMC iterations. *Dist* indicates the distance in kilometers between sites.

*P < 0.05 after 1000 genotype permutations.

### Historical demography results

**Fig. 4:**
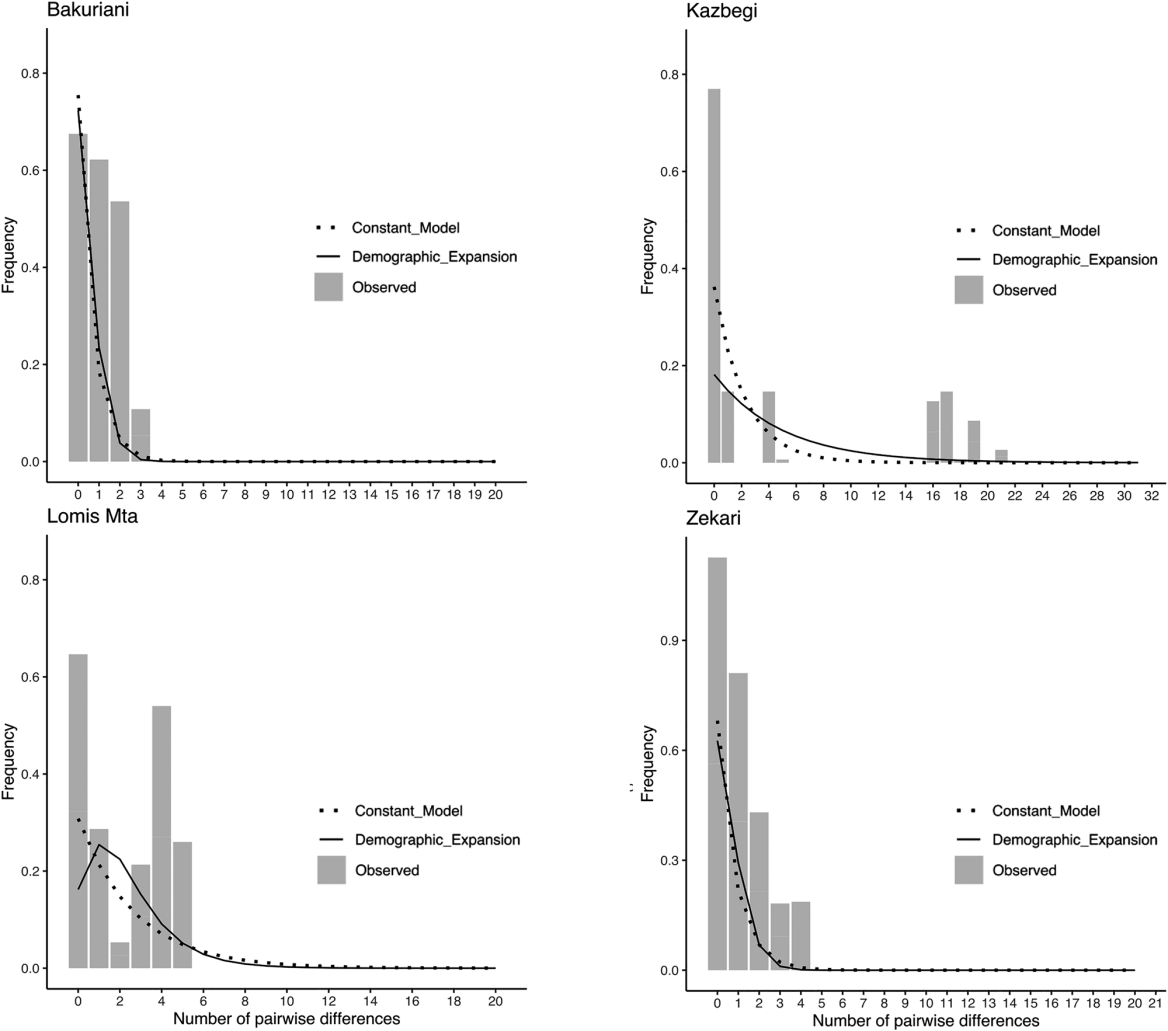
Distribution of COI observed pairwise nucleotide‐site differences and expected values for populations at equilibrium and for population expansions of wood tiger moth (*Arctia plantaginis*) males from four regions in Georgia, Caucasus.

The mitochondrial analyses showed a total of 10 haplotypes, with moderate to high haplotype diversity (Table 2). Most of the haplotypes were shared between samples from Bakuriani, Kazbegi, and Lomis Mta. Interestingly, samples from Zekari did not share haplotypes with the rest (Fig. 3D). Skewed unimodal mismatch distributions were observed for Zekari and Bakuriani, suggesting recent population expansions or bottlenecks. Lomis Mta showed a unimodal distribution, indicating a sudden expansion or a strong selective sweep, while Kazbegi had a multimodal distribution, indicative of a stable population size (Fig. 4). None of the raggedness indices (*r*) were significant (Table 2), indicating a relatively good fit between the observed data and the models. Summary statistics are presented in Table S2.

**Table 2:** Mitochondrial haplotype statistics and populations’ Lat: latitude, Lon: longitude, Elev: elevation in meters above sea level, *n*: number of samples sequenced, *h*: number of haplotypes, *Hd*: haplotype diversity, *r*: Harpending's raggedness index

Site	Lat	Lon	Elev	*n*	*h*	*Hd*	*r*
Bakuriani	41.69	43.52	2563	16	2	0.325	0.122
Kazbegi	42.66	44.58	2637	25	4	0.230	0.350
Lomis Mta	41.86	43.24	2154	25	5	0.676	0.082
Zekari	41.83	42.83	2236	37	2	0.315	0.120

### Genomic results

Approximately 47 million reads were aligned per sample, with 93% mapping efficiency, resulting in an average coverage of 8.7× per sample (Table S3). The genome‐wide expected heterozygosity was higher than the observed heterozygosity, leading to a negative inbreeding coefficient (*F*) across all samples (Table S3). This suggests an excess of heterozygotes, indicating that the Zekari population is not inbred. Regions with particularly low nucleotide diversity could not be clearly identified due to the relatively low and uniform nucleotide diversity observed across the genome (mean *π* = 0.0110, s.d. = 0.009, Fig. S2). However, we identified 13 genomic regions with notably low Tajima's *D* values (< −2), six of which contain genes homologous to *D. melanogaster* genes primarily involved in cellular energy regulation, glucose metabolism, and developmental processes (Table S4). We did not observe significantly high linkage disequilibrium (LD) within these 13 regions (Fig. S3).

## Discussion

In this study, we assess genetic variation of male wood tiger moths across part of the Caucasus mountain range and infer its potential links with differences in their orange‐red hindwing coloration. Using a combination of genetic and phenotypic approaches, we find geographical differences in the distribution of genetic diversity, and local similarities but regional differences in hindwing coloration. Moths from the Lesser Caucasus have predominantly large red wings and constitute mostly a single genetic population. Moths from the Greater Caucasus, in contrast, appear genetically isolated and are relatively small with orange hindwings. Below we discuss possible environmental, physiological, and geological processes that may help explain the associations (or lack thereof) between genetic/genomic patterns and geographical differences in hindwing size and coloration. Additionally, we outline directions for future research to better understand the lack of male‐limited color polymorphism in this area.

Our results indicate that moths from the Lesser Caucasus (Bakuriani, Lomis Mta, and Zekari) form a single panmictic population, as suggested by the microsatellite dataset (Fig. 3B, C and Table 1). Moths from these sites are predominantly red (Fig. 2A, B). While Lomis Mta and Zekari are situated within the same mountain range, Bakuriani is separated from them by the Borjomi Valley (Fig. 1), suggesting potential habitat discontinuity between these locations. Habitat discontinuity is often cited as a cause of gene flow disruption between populations (Temunović *et al.*, 2012; Ma *et al.*, 2018). However, despite this potential barrier, genetic connectivity appears to be maintained among these populations (Fig. 3B, C and Table 1). This connectivity is likely facilitated by adult male dispersal, as adult females have restricted flight capabilities, typically traveling only tens of meters, as indicated by mark‐recapture data (Gordon *et al.*, unpublished). In contrast, adult males can fly swiftly and disappear from sight (authors’ personal observations), likely contributing to the genetic homogeneity in the area.

Alternatively, dispersal may also occur during the larval stage, which lasts for almost a year, from approximately late June to mid‐July until mid‐May to early June (Ojala *et al.*, 2007; Galarza *et al.*, 2019). Given the prolonged larval stage and the high mobility of larvae observed in the laboratory, there is considerable potential for dispersal, at least between populations along the same mountain range, such as Lomis Mta and Zekari. However, data on larval dispersal remain limited, highlighting the need for further research on this aspect.

The genetic differences between the Lesser Caucasus and Greater Caucasus populations may also be influenced by geographic distance (Table 1). However, some of our previous studies show genetic connectivity between wood tiger moth populations across distances comparable to those between the Lesser and Greater Caucasus (Galarza *et al.*, 2014; Hegna & Mappes, 2014). Conversely, we have also observed genetic divergence between populations separated by much shorter distances (≈ 50 km) particularly when physical barriers such as water bodies, like the Gulf of Finland, are present (Galarza *et al.*, 2014; Murphy *et al.*, unpublished). While suitable habitats can facilitate genetic connectivity through stepping‐stone dispersal over relatively short distances, as seen between sites in the Lesser Caucasus (Table 1), the orography of the Caucasus region, characterized by rugged mountain ranges, deep valleys, and complex terrain, may act as a significant barrier to dispersal. This challenging topography could restrict movement and gene flow between populations, contributing to the observed genetic divergence between the Lesser and Greater Caucasus.

The differences in hindwing coloration between the Lesser and Greater Caucasus can be partly explained by differential selection pressure from bird predators, potentially driven by variations in the density of other co‐occurring aposematic Lepidopterans, such as red‐colored burnet moths (*Zygaenidae* spp.) and yellow‐colored scarlet tiger moths (*Callimorpha dominula*). In wood tiger moths, local differences in the frequency or density of warningly colored species are known to influence predator selection through mechanisms such as initial avoidance, generalization and learning (Nokelainen *et al.*, 2014; Rönkä *et al.*, 2018b). Furthermore, our research shows that local bird communities can create mosaic‐like selection patterns, favoring specific color morphs through positive frequency‐dependent selection (Rönkä *et al.*, 2020a). Therefore, the combination of ongoing gene flow within the Lesser Caucasus, limited connectivity with the Greater Caucasus, and potential differences in the abundance of other aposematic species may contribute to the higher frequency of red coloration observed in the Lesser Caucasus and the greater prevalence of orange coloration in the Greater Caucasus.

The predominance of orange hindwing coloration in the Greater Caucasus is intriguing, especially given that brighter colors generally enhance the effectiveness of warning signals (Joron, 2009; Briolat *et al.*, 2019). In the wood tiger moth, bird predation experiments show that females with more intense red hindwings experience fewer attacks from birds compared to those with orange hindwings (Lindstedt *et al.*, 2011). Moreover, birds learn to avoid artificial wood tiger moths with red wings significantly faster than those with white or yellow wings (Rönkä *et al.*, 2018a), and red moths tend to elicit more beak wiping than the other two morphs, a clear response of disgust indicating higher unpalatability (Gordon *et al.*, 2021). Thus, selection for a predominantly red coloration can be expected, as it appears to be a more efficient warning signal. However, the high prevalence of orange coloration in the Greater Caucasus suggests that other factors, possibly of historical origin, may be influencing this pattern.

The historical demographic profile of Kazbegi suggests a relatively stable population that has recently experienced a selective sweep (Fig. 4). In such case, selection may act on the available genetic variation rather than favoring the optimal phenotype. In contrast, populations in the Lesser Caucasus show evidence of recent or rapid population expansions (Fig. 4), which may have promoted greater genetic diversity and the persistence of the red coloration. These historical demographic differences could, at least in part, help explain why the suboptimal orange warning signal is predominantly present in the Greater Caucasus.

Historical adaptations may have also played a role in the larger body sizes and unique haplotypes observed in individuals from Zekari. Similar haplotype‐size associations in insects are often attributed to historical local adaptations (Mustafa *et al.*, 2015). Oxygen availability is a key factor in insect size, with historical atmospheric oxygen fluctuations correlating with body size changes in *Prodonata* and *Odonata* during periods like the late Oligocene and early Miocene, when the Caucasus mountains rose (Harrison & Lighton, 1998; Okajima, 2008; Callier & Nijhout, 2011). Some of the genes flagged by our analyses as potentially under selection may play a role in adaptation to hypoxic environments (Tabe S4). *Didum* is involved in ATPase activity and regulates processes such as mRNA localization and cell development (Krauss *et al.*, 2009). This regulation could help larvae compensate for low oxygen levels by optimizing cellular functions, thereby supporting more efficient growth. Similarly, *Glut1* facilitates glucose uptake and homeostasis, which are critical for maintaining energy production through glycolysis under hypoxic conditions (Kauffman & DiAngelo, 2024). Additionally, *Oseg4* regulates the canonical Wnt signaling pathway, which is directly involved in body patterning, cell growth, and differentiation (Balmer *et al.*, 2015; Lee *et al.*, 2018). This pathway is key to processes like body axis formation and tissue regeneration, both of which can influence organism size and morphology. Hence, the larger size of the individuals from Zekari, and their unique haplotypes, may represent a relict adaptation to atmospheric oxygen variations during the rise of the Lesser Caucasus.

The above hypothesis needs to be corroborated by functional expression studies and raises the question of why individuals from the neighboring Lomis Mta and Bakuriani do not possess unique haplotypes and are smaller, despite ongoing gene flow and similar historical demographics. Likely, additional pressures, such as microclimatic conditions, unique ecological interactions, or differences in food sources at Zekari, may be influencing these patterns. To make more robust inferences, vegetation surveys and whole‐genome comparisons across sites are needed. Nonetheless, our findings provide a valuable starting point for future research aimed at understanding the genetic basis of size variation in the region.

### Conclusion and further directions

Our examination of genetic and phenotypic variations across different geographical regions helps clarify historical and contemporary processes that may contribute to the observed variation in male wood tiger moth coloration within Georgia. It is important to note that the genetic basis of the distinctive male orange‐red coloration remains unknown. While we have made substantial progress in elucidating the genetic basis of color polymorphism in yellow and white males through pedigree analyses (Nokelainen *et al.*, 2022), and CRISPR knockouts (Brien *et al.*, 2023) establishing that the yellow‐white polymorphism follows Mendelian inheritance within a one‐locus two‐allele model, and that a single gene duplication produces the white coloration, we have yet to explore the genetic mechanisms underlying the orange‐red coloration observed in red males in the Caucasus region. Future studies are needed to investigate these mechanisms, which may involve different genetic pathways or regulatory elements, to provide a more comprehensive understanding of the genetic architecture driving color variation in this species.

## Disclosure

The authors declare no conflict of interest.

## Supporting information




**Table S1** Nuclear microsatellite panel for wood tiger moths (*Arctia plantaginis*) from Georgia.
**Table S2** *Cytochrome oxidase subunit I* (*COI*) gene fragments of wood tiger moths (*Arctia plantaginis*) from Georgia.
**Table S3** Mapping and heterozygosity statistics of whole‐genome sequencing reads from *Arctia plantaginis* males from Georgia, Caucasus.
**Table S4** Summary of genomic windows and associated genes.

**Fig. S1** Male wood tiger moth (*Arctia plantaginis*) color metrics in forewing hue (A), saturation (B), and brightness (C), as well as hindwing hue (D).

**Fig. S2** Genome‐wide nucleotide diversity (pi) and Tajima's *D* values from wood tiger moth (*Arctia plantaginis*) males from Zekari, Georgia, Caucasus.

**Fig. S3** Linkage disequilibrium across (*R* ^2^)10 Kb windows in 13 genomic regions of male wood tiger moth (*Arctia plantaginis*) from Zekari, Georgia, Caucasus.
